# Strain echocardiography predictors in patients with concomitant cardiac amyloidosis and aortic stenosis: a cross-sectional study

**DOI:** 10.1186/s12872-024-04415-8

**Published:** 2024-12-20

**Authors:** Samira Jafarisis, Shahab Masoumi, Naser Khezerlouy-Aghdam, Kia Seyed Toutounchi, Amirreza Jabbaripour Sarmadian, Sina Hamzehzadeh, Akram Shariati, Razieh Parizad, Venus Shahabi Rabori

**Affiliations:** 1 Cardiovascular Research Center, Tabriz University of Medical Sciences, Daneshgah Street, Tabriz, Eastern Azerbaijan Iran; 2 Department of Cardiology, School of Medicine, Urmia University of Medical Sciences, Urmia, Iran; 3 Cardiology Department, Royal Albert Edward Infirmary, WWL NHS Trust, Wigan, UK

**Keywords:** Amyloidosis, Aortic stenosis, Echocardiography, Strain

## Abstract

**Background:**

Concomitant cardiac amyloidosis (CA) and aortic stenosis (AS) may be mistaken for isolated AS, potentially impacting the treatment strategy and patient’s prognosis. Therefore, it is crucial to distinguish between these conditions, as failure to promptly diagnose CA may lead to considerable complications. The aim of this study is to investigate the diagnostic value of strain predictors in patients with concomitant CA and AS compared to isolated AS.

**Methods:**

Forty-two patients with severe AS suspected of concomitant CA based on a comprehensive clinical evaluation were selected to undergo ^99^mTc-DPD scintigraphy. Those showing Perugini grade 2 or 3 tracer uptakes without evidence of monoclonal gammopathy were diagnosed with CA and underwent speckle-tracking echocardiography. Furthermore, strain analysis was performed to evaluate myocardial deformation, with a focus on detecting apical sparing and reduction in bull’s eye mapping, resulting in the characteristic “cherry on top” sign.

**Results:**

Eight patients were diagnosed with CA, representing 19.0% of those suspected of concomitant CA and 7.8% of the overall cohort with severe AS. AF arrhythmia was significantly more frequent in these patients compared to those with isolated AS. Echocardiography findings revealed that E/E’ ratio and RALS were significantly higher in patients with concomitant CA, while GLS and mean basal LS were significantly lower in this group. The “cherry on top” sign was detected in 19 patients (45.2%), present in 100% of those with concomitant CA and AS, versus 32.4% in isolated AS cases (*P* = 0.04). This sign demonstrated a sensitivity of 100% and a specificity of 67.6% for predicting concomitant CA and AS.

**Conclusions:**

In conclusion, the “cherry on top” sign was significantly more prevalent in patients with concomitant CA and AS, compared to those with isolated AS, demonstrating a sensitivity of 100% and a specificity of 67.6% for predicting concomitant CA. Moreover, RALS and E/E’ ratios were significantly higher in patients with concomitant CA, whereas GLS and mean basal LS were significantly lower in this group.

## Introduction

Cardiac amyloidosis (CA) is a rare myocardial disorder characterized by the extracellular infiltration of insoluble amyloid fibrils throughout the cardiac chambers, resulting in ventricular wall thickening, cardiac remodeling, valvular heart disease (VHD), diastolic dysfunction, arrhythmias, heart failure, and subsequent restrictive cardiomyopathy [1–5]. It can be classified into several subtypes based on the accumulated fibrils, with over 95% of cases being amyloid light chain cardiac amyloidosis (AL-CA), resulting from monoclonal plasma cell-derived immunoglobulin deposition and amyloid transthyretin cardiac amyloidosis (ATTR-CA), resulting from liver-derived plasma protein deposition with further divided into wild-type ATTR-CA (ATTRwt-CA) and variant/hereditary ATTR-CA (ATTRv-CA) amyloidosis [2, 3]. It has a 9% prevalence among hypertrophic cardiomyopathies [6], also a 4–29% incidence has been reported among all population, which increases with older age [7].

As mentioned, VHD is one of the critical complications of CA, contributing to worsened clinical outcomes and prognosis, commonly presenting with aortic stenosis (AS), mitral regurgitation (MR), and tricuspid regurgitation (TR). On the other hand, AS is recognized as the most prevalent form of VHD in the general population, leading to left ventricular (LV) wall thickening and diastolic dysfunction, features also observed in CA [8]. Therefore, concomitant CA and AS may be mistaken for isolated AS, potentially impacting the treatment strategy and patient’s prognosis. Hence, it is crucial to distinguish between these conditions, as failure to promptly diagnose CA may lead to heart failure, atrial fibrillation (AF), thromboembolic events, and considerable mortality [7].

Although endomyocardial biopsy serves as the gold standard for confirming the diagnosis of CA, its invasive nature and the need for high expertise result in its less frequent utilization. Instead, alternative paraclinical modalities such as electrocardiography (ECG), cardiac magnetic resonance (CMR), radionuclide bone scintigraphy (RBS), and echocardiography are often preferred. These methods, combined with clinical assessment and biomarker testing, contribute to a comprehensive diagnostic approach for CA [2, 4]. As the importance of CA timely diagnosis has been discussed above, therefore, ESC 2021 guidelines for HF have dedicated a guideline for CA management, concluding that HF with LV thickness over 12 mm in > 65-year-old-patients or patients with other clinical and paraclinical red flags for CA (as mentioned above) should undergo serum/urine protein assay and RBS [9].

In these patients, conventional echocardiography, the most commonly used imaging modality in cardiology [10, 11], typically reveals thickening of the ventricular walls, the septal wall (exceeding 12 mm), and the aortic valve. Other common findings include atrial enlargement, granular myocardial sparkling, and elevated filling pressures. In addition, a normal to high ejection fraction (EF) in the presence of heart failure is highly suggestive of CA. However, coexisting conditions such as VHD or chronic hypertension (HTN) can affect the reliability of these features [12].

Therefore, more advanced parameters such as strain and strain rate have been proposed to provide quantitative assessments of myocardial deformation and contractility. Strain measures the percentage change in myocardial length throughout the cardiac cycle, while strain rate reflects the rate of global or regional myocardial deformation [13]. Patients with CA typically exhibit impaired longitudinal strain (LS), with the apical segments remaining relatively unaffected - a pattern commonly referred to as the “cherry on top” sign. This is characterized by an apical LS-to-mid and basal-LS ratio greater than 1, which serves as a key diagnostic marker for the condition [14, 15]. However, more studies are needed in this field to be used in daily practice, especially in more complicated patients. Therefore, in this study, we aimed to investigate the diagnostic value of strain predictors in patients with concomitant CA and severe AS compared to isolated severe AS.

## Methods and materials

### Methods

#### Study design and setting

This study was designed and conducted as a case-control study over four years between May 2018 and May 2022 in the echocardiography laboratory of the Tabriz Shahid Madani Heart Center, to investigate the diagnostic value of strain predictors in patients with concomitant CA and severe AS compared to isolated severe AS.

#### Participants (inclusion and exclusion criteria)

Among all patients over the age of 18 who were referred to our center and underwent echocardiography, those diagnosed with severe AS defined by aortic valve area (AVA) ≤ 1, transvalvular pressure gradient (TVPG) ≥ 40 mmHg, or aortic transvalvular velocity (V_max_) ≥ 4 m/s were studied [16]. Patients with pseudo-AS, poor acoustic window, prior coronary artery disease, regional wall motion abnormality compatible with ischemic heart disease (IHD), bicuspid aortic valve, and rheumatic valve disease were excluded from the study. Then, patients with severe AS suspected of concomitant CA based on the comprehensive clinical assessment, ECG, conventional echocardiography, or any evidence of systemic amyloidosis based on diagnostic criteria were selected.

#### Study protocol

Of the 102 included patients, those with severe AS suspected of concomitant CA based on a comprehensive clinical evaluation were selected for further assessment. Suspicion was raised by presentations such as carpal tunnel syndrome (CTS), lumbar spinal stenosis, deafness, early pacemaker implantation, rupture of the bicep tendon, polyneuropathy, unexplained weight loss, heart failure with preserved EF, AF arrhythmia, or autonomic dysfunction, as well as findings from ECG, conventional echocardiography, or evidence of systemic amyloidosis including the presence of monoclonal light chains in serum or urine, plasma cell dyscrasia in bone marrow biopsy, or positive tissue biopsy confirming light chain amyloid deposition in any organ. As a result, 42 patients were selected to undergo ^99m^Tc-3,3-Diphosphono-1,2-Propanodicarboxylic acid (^99m^Tc-DPD) scintigraphy. Those showing Perugini grade 2 or 3 tracer uptakes without evidence of monoclonal gammopathy were diagnosed with CA and underwent speckle-tracking echocardiography (STE). Furthermore, strain analysis was performed on suspected patients to evaluate myocardial deformation, with a focus on detecting apical sparing of LS compared to the mid and basal segments. This analysis aimed to detect the reduction in bull’s eye mapping, resulting in the characteristic “cherry on top” sign.

### Materials

#### Echocardiography

##### Conventional echocardiography

All patients referred to our center underwent transthoracic echocardiography (TTE) utilizing a Philips^®^ EPIQ 7 ultrasound system to detect severe AS according to the mentioned criteria [17]. Moreover, other parameters were analyzed in suspected patients including EF, LV wall thickness, systolic pulmonary artery pressure (sPAP), aortic valve area index (AVAI), aortic valve area by continuity equation (AVA-CE), aortic valve pressure gradient (AV-PG), and aortic valve mean gradian (AV-MG). QLab software was used for all mathematical analyses.

##### Doppler echocardiography

All patients with severe AS suspected of concomitant CA underwent Doppler echocardiography utilizing a Philips^®^ EPIQ 7 ultrasound system to evaluate cardiac wall motion velocity and distinguish contractility from passive wall motions. Therefore, E-wave velocity, E/A ratio, E-wave deceleration time, and E/E’ ratio were analyzed.

##### STE

All patients with severe AS suspected of concomitant CA underwent STE utilizing a Philips^®^ EPIQ 7 ultrasound system, which tracks the movement of naturally occurring acoustic markers or “speckles”, within the heart tissue to assess global and regional myocardial function. By measuring the distance between speckles, the system evaluates global longitudinal strain (GLS) as well as regional strain, including basal, mid, and apical LS, to detect subclinical LV dysfunction. The relative apical longitudinal strain (RALS) was analyzed using the following formula: RALS = Mean Apical LS / (Mean Basal LS + Mean Mid LS). Moreover, the bull’s eye mapping of patients was studied to detect “cherry on top” sign [14, 15].

#### ECG

All referred patients underwent ECG to detect features indicative of prior IHD, LV wall thickening, arrhythmias - particularly AF, pseudo-infarct patterns, and low voltage readings.

#### Scintigraphy

All patients with severe AS suspected of concomitant CA underwent scintigraphy imaging technique to diagnose CA. Following the intravenous administration of the ^99m^Tc-DPD radiotracer, patients underwent both planar imaging and single-photon emission computed tomography (SPECT). Cardiac uptake was assessed and graded in comparison to rib bone uptake. According to the Perugini scoring system, the grades are defined as follows: 0 = no cardiac uptake, 1 = cardiac uptake less than that of rib bone (mild), 2 = cardiac uptake equal to that of rib bone (moderate), and 3 = cardiac uptake greater than that of rib bone (severe) [18]. Studies indicate that a positive scintigraphy result (grade 2 or 3 on the Perugini scoring system) in the absence of monoclonal gammopathy on serum and urine analysis holds diagnostic value even without histopathologic confirmation, especially for ATTR-CA [19].

### Statistical analysis

Data were collected and analyzed by SPSS software version 26. Kolmogorov–Smirnov test was used to evaluate the normal distribution of quantitative parameters. For continuous variables, mean and standard deviation were reported, and for nominal and ordinal variables, frequencies and percentages were reported. For analysis of the parameters between groups, the independent sample t-test was used for normally distributed data, and the Mann–Whitney U test was used for not normally distributed quantitative data. A Chi-squared test was used to compare qualitative parameters. In all tests, a P-Value less than 0.05 was considered a significant difference.

### Ethics

This study was approved by the Tabriz University of Medical Sciences ethics committee with the approval number IR.TBZMED.REC.1400.853 on 29/11/2021. All patients signed and accepted informed consent papers; their participation in this study and the data they provided are completely confidential.

## Results

As previously noted, 102 patients with severe AS were evaluated, of whom 42 (41.2%) were suspected of having concurrent CA based on comprehensive clinical assessments and were subsequently enrolled in the study. The mean age of these patients was 62.02 ± 13.15 years, comprising 27 males (64.3%) and 15 females (35.7%). Using ^99m^Tc-DPD scintigraphy, amyloidosis was detected in 8 patients, representing 19.0% of those suspected of concomitant CA and 7.8% of the overall cohort with severe AS.

As shown in Table 1, AF arrhythmia was significantly more common in patients with concomitant CA compared to those with isolated AS (P-Value = 0.016); however, no significant differences were found between the two groups in terms of age, gender, history of HTN, diabetes mellitus (DM), CTS, New York Heart Association (NYHA) functional classification, systolic blood pressure (SBP), diastolic blood pressure (DBP), or low voltage ECG findings. Regarding TTE findings, E/E’ ratio and RALS were significantly higher in patients with concomitant CA (P-Values = 0.044 and 0.039, respectively). In addition, GLS and mean basal LS were significantly lower in this group of patients (P-Values = 0.045 and 0.043, respectively). No significant differences were found between the two groups in terms of EF, left ventricular hypertrophy (LVH), sPAP, AVAI, AVA-CE, AVA-PG, AV-MG, E-wave velocity, E/A ratio, E-Wave deceleration time, mean apical LS, and mean mid-cavity LS, as shown in Table 2.

**Table 1:** Demographic and clinical characteristics of patients

Parameters			All patients	Concomitant CA and AS	Isolated AS	*P*-Value
**Number of Patients**			42	8 (19.0%)	34 (81.0%)	0.514
**Age**			62.02 ± 13.15	56.00 ± 2.82	62.32 ± 13.40	0.336
**Gender**	**Male**		27 (64.3%)	4 (50.0%)	23 (67.6%)	0.433
	**Female**		15 (35.7%)	4 (50.0%)	11 (32.4%)	0.834
**HTN**			24 (57.1%)	4 (50.0%)	20 (58.8%)	0.322
**DM**			13 (31.0%)	0 (0.0%)	13 (38.2%)	0.388
**CTS**			4 (9.5%)	0 (0.0%)	4 (11.8%)	0.769
**NYHA Functional Classification**		**1**	2 (4.8%)	0 (0.0%)	2 (5.9%)	0.918
		**2**	16 (38.1%)	4 (50.0%)	12 (35.3%)	
		**3**	17 (40.5%)	4 (50.0%)	13 (38.2%)	
		**4**	7 (16.7%)	0 (0.0%)	7 (20.6%)	
**SBP**			131.17 ± 14.00	125.00 ± 7.07	131.65 ± 14.13	0.666
**DBP**			73.83 ± 15.08	70.00 ± 14.04	74.18 ± 15.43	0.716
**Low Voltage ECG**			11 (26.2%)	1 (12.5%)	10 (29.4%)	0.638
**AF**			6 (14.3%)	4 (50.0%)	2 (5.9%)	0.016*

**Table 2:** TTE findings of patients including conventional echocardiography, Doppler echocardiography, and STE

TTE Findings		All patients	Concomitant CA and AS	Isolated AS	*P*-Value
**Number of Patients with EF < 50%**		17 (40.5%)	4 (50.0%)	13 (38.2%)	0.527
**LVH**	**Mild LVH**	21 (50.0%)	4 (50.0%)	17 (50.0%)	0.892
	**Moderate LVH**	15 (35.7%)	4 (50.0%)	11 (32.4%)	
	**Severe LVH**	4 (9.5%)	0 (0.0%)	4 (11.8%)	
**sPAP**		35.90 ± 3.03	44.00 ± 1.41	33.90 ± 1.50	0.372
**AVAI**		0.65 ± 0.15	0.80 ± 0.14	0.62 ± 0.21	0.281
**AVA-CE**		0.95 ± 0.35	1.09 ± 0.15	0.92 ± 0.24	0.354
**AV-PG**		73.80 ± 7.79	73.50 ± 19.09	73.90 ± 6.99	0.981
**AV-MG**		43.90 ± 5.32	42.50 ± 7.67	44.21 ± 6.49	0.887
**E-Wave Velocity**		0.76 ± 0.21	0.75 ± 0.06	0.76 ± 0.22	0.953
**E/A Ratio**		0.93 ± 0.40	1.02 ± 0.52	0.91 ± 0.35	0.699
**E-Wave Deceleration Time**		224.03 ± 93.39	201.50 ± 44.50	222.90 ± 45.90	0.678
**E/E’ Ratio**		6.00 ± 7.20	12.00 ± 1.20	4.60 ± 0.80	0.044*
**GLS**		16.00 ± 4.70	14.40 ± 1.97	16.46 ± 4.18	0.045*
**Mean Apical LS**		19.70 ± 2.01	21.70 ± 0.20	19.20 ± 1.10	0.783
**Mean Mid-Cavity LS**		14.23 ± 4.50	11.44 ± 1.61	14.78 ± 1.50	0.200
**Mean Basal LS**		14.70 ± 2.30	9.10 ± 1.41	16.20 ± 1.50	0.043*
**RALS**		0.68 ± 0.05	1.05 ± 0.04	0.61 ± 0.06	0.039*
**“Cherry on Top” Pattern**		19 (45.2%)	8 (100.0%)	11 (32.4%)	0.04*
**LVDD**	**G1**	23 (54.8%)	4 (50.0%)	19 (55.9%)	0.199
	**G2**	13 (31.0%)	2 (25.0%)	11 (32.4%)	
	**G3**	6 (14.3%)	2 (25.0%)	4 (11.8%)	

The “cherry on top” sign was detected in 19 patients (45.2%), being present in 100% of those with concomitant CA and AS, compared to 32.4% of patients with isolated AS (P-Value = 0.04). There were no significant differences between the two groups regarding left ventricular diastolic dysfunction (LVDD), as shown in Table 2 (P-Value = 0.199).

All patients exhibiting the “cherry on top” sign had a RALS level above 1.00; however, no significant differences were found between those with the “cherry on top” sign and confirmed CA, and those with the sign but without CA (P-Value = 0.10). The “cherry on top” sign demonstrated a sensitivity of 100% and a specificity of 67.6% for predicting concomitant CA and AS, with a positive predictive value of 42.1% and a negative predictive value of 100%. The positive likelihood ratio was 3.08, and the negative likelihood ratio was 0.

## Discussion

This study aimed to investigate the accuracy of strain echocardiography in detecting CA in patients with severe AS, with a focus on the relative sparing of the apical segments - commonly known as the “cherry on top” sign in bull’s eye mapping. This pattern demonstrated a sensitivity of 100% and a specificity of 67.6% in identifying CA.

There are controversial theories regarding the pathophysiology of concomitant CA and AS, with some suggesting that AS may induce CA by imposing pressure overload that affects myocardial remodeling, potentially exacerbating or triggering transthyretin amyloid deposition. Conversely, other theories suggest that amyloid deposition may precede and contribute to aortic valve changes. It should be noted that AS is more reported in cases of ATTR-CA than AL-CA, with age being a significant contributing factor [7, 20]. Based on our comprehensive literature review, both clinical and paraclinical findings are essential for screening suspected CA. Studies suggest that older male patients with severe AS and a history of CTS, lumbar spinal stenosis, deafness, early pacemaker implantation, bicep tendon rupture, polyneuropathy, unexplained weight loss, elevated cardiac biomarkers (such as pro-BNP and troponin), AF arrhythmia, heart failure with preserved EF, or autonomic dysfunction should be considered for CA screening, especially ATTR-CA. In addition, findings from ECG, echocardiography, or evidence of systemic amyloidosis, such as monoclonal light chains in serum or urine, plasma cell dyscrasia in bone marrow biopsy, or positive tissue biopsy confirming light chain amyloid deposition in any organ, further support the need for screening [21, 22]. The incidence of ATTR-CA in patients with AS has been reported in a few studies, ranging from 4 to 29%, with factors such as age and gender influencing these rates [7]. Considering the mentioned features, 42 patients (41.2% of the overall cohort with severe AS) were suspected and screened for concomitant CA and AS. Of these, 8 patients (7.8% of the overall cohort with severe AS and 19% of patients with suspected CA) were diagnosed with CA based on scintigraphy findings, with no significant differences observed in age or gender between these patients and isolated AS patients. The prevalence of CA in AS patients has been reported in several studies ranging from 8 to 22% [23, 24], which are conformable with our finding.

Common echocardiographic findings in these patients, such as increased LV wall thickness and both systolic and diastolic dysfunction, are also seen in other conditions like isolated AS and HTN [12]. In a review study conducted by Rachele M. et al. useful echocardiographic parameters to identify patients with severe AS are AVA, TVPG, aortic valve velocity, acceleration time to ejection time ratio and etc [25]. By using these parameters, we selected severe AS cases and assessed the presence of CA in them.

In our study, no significant differences were found between patients with concomitant CA and those with isolated AS in terms of LV wall thickness or systolic dysfunction, as indicated by decreased EF. Moreover, Doppler echocardiographic parameters such as E-wave velocity, E/A ratio, E-wave deceleration time, and E/E’ ratio were analyzed to assess LV diastolic dysfunction [26]. In these patients, higher E-wave velocity, E/A ratio, and E/E’ ratio, as well as a shorter deceleration time (< 200 ms) are expected due to the stiffening of the heart muscle and elevated filling pressures [27]. However, in our study, the only significant difference observed between the two groups was in the E/E’ ratio. In a study by Castano et al. [24], patients with ATTR-CA showed a significantly higher LV mass index, thicker interventricular septum, and lower stroke volume index. Additionally, they had significant systolic dysfunction, as indicated by lower EF, reduced myocardial contraction fraction, and a decreased average lateral and septal mitral annular tissue Doppler S’. They also showed advanced diastolic dysfunction, with a higher E/A ratio and shorter E-wave deceleration time, reflecting impaired filling dynamics.

In addition to the mentioned parameters, global and regional longitudinal strains are evaluated using STE to detect myocardial fiber deformation and predict subclinical LV dysfunction. Therefore, some experts recommend that GLS analysis be routinely performed alongside EF assessment to provide a more comprehensive evaluation of LV systolic function, with suggested normal values of 17–24% for males and 18–26% for females [28–30]. In our study, the mean GLS in patients with concomitant CA was 14.40 ± 1.97, which was significantly lower compared to the group with isolated AS.

It should be noted that in patients with CA, LS is unevenly affected across the LV, with significant impairment in the mid and basal segments but relative preservation in the apex, a pattern known as “apical sparing” that is particularly characteristic of ATTRwt-CA and gives the appearance of a “cherry on top” sign in bull’s eye mapping [14, 27]. Three hypotheses may explain the presence of this pattern: reduced amyloid deposition at the apex, increased sensitivity of the basal segments to necrosis, and greater diversity of myocytes in the apex [31]. Phelan et al. [32] reported a RALS cutoff of 1.0, with a sensitivity of 93% and specificity of 82% for differentiating CA from control patients with LVH. In our study, the mean RALS was 1.05 ± 0.04, which was significantly higher than the group with isolated AS. In another study, Robin et al. [33] reported a RALS cutoff of 1.0, with a sensitivity of 88% and specificity of 68% for differentiating ATTRwt-CA among patients with severe AS. However, Castano et al. [24] reported no significant differences regarding RALS between patients with ATTR-CA compared to the group with isolated AS. Similarly, Nitsche et al. [34] reported no significant differences regarding RALS between patients with concomitant CA compared to the group with isolated AS, even with a cutoff of 1.0.

In our study, the “cherry on top” sign demonstrated a sensitivity of 100% and a specificity of 67.6% for predicting concomitant CA and AS, with a positive predictive value of 42.1% and a negative predictive value of 100%. In 11 patients with isolated AS (32.4%), the “cherry on top” sign was also observed, aligning with findings from other studies that report apical sparing in patients with isolated AS [35–38].

Patients with cardiac amyloidosis had a greater risk of AF in our study. It is known that due to amyloid deposition in the myocardium, the cardiac conduction system is interrupted, causing cardiac arrhythmias, such as atrial fibrillation [39, 40]. Additionally atrial enlargement and autonomic dysfunction worsens the risk of AF prevalence as the disease progresses to advance stages [41].

To manage severe aortic stenosis (AS), aortic valve replacement (AVR) procedures, including surgical AVR (SAVR) and transcatheter AVR (TAVR), have been shown to significantly reduce LV mass by alleviating LV pressure overload, as observed in post-surgical echocardiographic assessments [38, 42]. However, in cases of AS complicated by CA, careful evaluation is essential to determine whether surgical or medical management is more appropriate, with the primary goal being to relieve the stenosis while simultaneously initiating specific treatment for CA. Recent studies suggest that TAVR achieves this more effectively than SAVR, offering better outcomes and lower mortality rates in this patient population [38]. 

### Limitations

The most important limitation of our study was that the diagnosis of CA was based on scintigraphy rather than endomyocardial biopsy, which is unable to accurately differentiate CA types. However, since a positive scintigraphy result (grade 2 or 3 on the Perugini scoring system) in the absence of monoclonal gammopathy on serum and urine analysis holds diagnostic value even without histopathologic confirmation, especially for ATTR-CA [19], we hypothesize that our findings are more compatible of this kind of CA. Another limitation was the sample size, as our study was a single-center study and given the rarity of cardiac amyloidosis; we included all eligible patients over a four-year period, resulting in a total of 102 patients with severe aortic stenosis, of whom 42 were suspected of having concurrent cardiac amyloidosis. It is recommended to perform further studies with a larger sample size and with categorization by CA type.

## Conclusion

In conclusion, the “cherry on top” sign was significantly more prevalent in patients with concomitant CA and AS, compared to those with isolated AS, demonstrating a sensitivity of 100% and a specificity of 67.6% for predicting concomitant CA. Moreover, RALS and E/E’ ratios were significantly higher in patients with concomitant CA, whereas GLS and mean basal LS were significantly lower in this group.

## Data Availability

Data is provided within the manuscript.

## Abbreviations

^99m^Tc-DPD: ^99m^Tc-3,3-diphosphono-1,2-propanodicarboxylicacid
AF: Atrial fibrillation
AL-CA: Amyloid light chain cardiac amyloidosis
AS: Aortic stenosis
ATTR-CA: Amyloid transthyretin cardiac amyloidosis
ATTRv-CA: Variant amyloid transthyretin cardiac amyloidosis
ATTRwt-CA: Wild-type amyloid transthyretin cardiac amyloidosis
AVA: Aortic valve area
AVA-CE: Aortic valve area by continuity equation
AVAI: Aortic valve area index
AV-PG: Aortic valve pressure gradient
AV-MG: Aortic valve mean gradian
CA: Cardiac amyloidosis
CMR: Cardiac magnetic resonance
CTS: Carpal tunnel syndrome
DBP: Diastolic blood pressure
DM: Diabetes mellitus
ECG: Electrocardiography
EF: Ejection fraction
GLS: Global longitudinal strain
HTN: Hypertension
IHD: Ischemic heart disease
LS: Longitudinal strain
LV: Left ventricular
LVDD: Left ventricular diastolic dysfunction
LVH: Left ventricular hypertrophy
MR: Mitral regurgitation
NYHA: Functional classification: new york heart association functional classification
RALS: Relative apical longitudinal strain
RBS: Radionuclide bone scintigraphy
SBP: Systolic blood pressure
sPAP: Systolic pulmonary artery pressure
SPECT: Single-photon emission computed tomography
STE: Speckle tracking echocardiography
TR: Tricuspid regurgitation
TTE: Transthoracic echocardiography
VHD: Valvular heart disease
TVPG: Transvalvular pressure gradient
AVR: Aortic valve replacement
SAVR: Surgical avr
TAVR: Transcatheter avr
